# Spontaneous Regression of Intracranial Aneurysms—Case Report and Systematic Review of the Literature

**DOI:** 10.3390/brainsci15050488

**Published:** 2025-05-06

**Authors:** Kristina Catalano, Lukas Andereggen, Gerrit A. Schubert, Serge Marbacher, Basil E. Grüter

**Affiliations:** 1 Department of Neurosurgery, Kantonsspital Aarau, 5001 Aarau, Switzerland; kristina.catalano@ksa.ch (K.C.); lukas.andereggen@ksa.ch (L.A.); gerrit.schubert@ksa.ch (G.A.S.); serge.marbacher@ksa.ch (S.M.); 2 Department of Neurosurgery, Kantonsspital Aarau, University of Bern, 3012 Bern, Switzerland; 3 Department of Neurosurgery, RWTH Aachen University, 52062 Aachen, Germany; 4 Institute of Neuroradiology, Department of Radiology, Kantonsspital Aarau, 5001 Aarau, Switzerland; 5 Department of Neurosurgery and Neuroradiology, HOCH Health Ostschweiz, 9000 St. Gallen, Switzerland

**Keywords:** intracranial aneurysm, spontaneous regression

## Abstract

**Background/Objectives**: The natural course of intracranial aneurysms (IAs) remains unclear. Many of them remain stable over time and few experience patterns of growth. The spontaneous regression of IAs without any microsurgical or endovascular treatment is a very rare phenomenon. This paper reports the case of a 56-year-old female who experienced spontaneous regression of her IA. Furthermore, it contains a systematic literature review to explore reported cases of spontaneous IA regression. **Methods**: The case of a 56-year old female patient who presented with an anterior communicating artery (ACom) IA that thrombosed spontaneously after 108 months follow-up is reported. Additionally, a systematic literature search was conducted using the Medline database to identify reported cases. **Results**: The IA showed spontaneous regression without any surgical or endovascular intervention. We identified 33 articles describing IAs with spontaneous regression. Reported reasons for spontaneous IA thrombosis included (1) anatomical factors like narrow aneurysmal necks; (2) coagulation pathway modifications, including antifibrinolytic activity that promotes thrombosis; and (3) hemodynamic changes such as altered blood flow dynamics and external vascular compression. These findings suggest that spontaneous regression, while rare and unpredictable, can be associated with distinct physiological and anatomical conditions. **Conclusions**: The spontaneous regression of IAs is an extremely rare phenomenon. It cannot reliably be predicted and may be associated with changes in the hemodynamic situation, specific anatomical constellations, or coagulation pathways.

## 1. Introduction

The spontaneous regression of intracranial aneurysms (IAs) is an extremely rare phenomenon that challenges traditional paradigms in cerebrovascular management. While most IAs remain stable or grow gradually, some cases exhibit spontaneous thrombosis without intervention [1], raising questions about the factors driving such outcomes.

Despite advances in imaging technologies that have improved detection rates, the natural history of IAs remains incompletely understood. The unpredictability of IA behavior complicates management decisions, particularly for smaller or incidentally detected IAs, where the risks of intervention must be weighed against the likelihood of rupture with caution [2].

The socio-economic burden associated with IA management is substantial. Costs arise from initial diagnostic workups, including advanced imaging, and extend through long-term follow-up, intervention, and rehabilitation for patients suffering from rupture-related complications [3]. Studies suggest that follow-up imaging and monitoring, particularly for unruptured IAs managed conservatively, constitute a significant portion of this burden [4]. Moreover, the emergence of conservative management strategies necessitates guidelines for determining follow-up intervals, monitoring criteria, and indications for therapeutic intervention. The lack of standardized protocols complicates healthcare planning and cost control while raising concerns about patient safety and outcomes [5,6,7]. These factors underscore the importance of understanding the natural history of IAs and the implications of both conservative and interventional management approaches [8,9].

This study presents a unique case of a 56-year-old female with spontaneous regression of an anterior communicating artery (ACom) aneurysm over a nine-year follow-up. Additionally, a systematic review was conducted to identify similar cases and potential contributing factors and explanatory mechanisms and their implications for clinical practice. IA regression is influenced by various factors, including hemodynamic stress and changes in intra-aneurysmal blood flow dynamics, which can alter endothelial function and promote thrombosis within the IA sac [10,11]. Further proposed mechanisms include external compression from surrounding tissues and alterations in coagulation pathways leading to thrombosis [12]. However, the rarity of regression and its largely unpredictable nature challenge our ability to develop reliable predictive models, necessitating detailed studies and longitudinal monitoring [8,9].

## 2. Materials and Methods

The study was approved by our institutional review board and the local ethics commission (EKNZ Nr. 2020-02249).

### 2.1. Definition of Spontaneous Regression

For the purpose of this review, we define “spontaneous regression” as a reduction in the size or complete thrombosis of an IA confirmed by at least two imaging modalities or serial imaging over a minimum of six months, occurring without any surgical or endovascular intervention. This definition excludes regression following flow-diversion, clipping, coiling, or inflammation-related pseudoaneurysm resolution.

Inclusion criteria for literature review included documented imaging at baseline and follow-up, absence of interventional treatment, and evidence of aneurysm size reduction or thrombosis. Exclusion criteria were post-treatment changes (e.g., residual necks), mycotic or traumatic aneurysms, and cases without clear imaging-based documentation.

### 2.2. Case Report

We present a detailed case report of a 56-year-old female with spontaneous IA thrombosis following a diagnosis of right-sided temporal intracranial bleeding. All clinical data were extracted from her medical records, including medical imaging. We obtained a written consent regarding the use of patient data for research purposes. The case report was structured in compliance with the CARE Guidelines to ensure transparency and the reproducibility of the presented findings [13].

### 2.3. Literature Review

**Figure 1:**
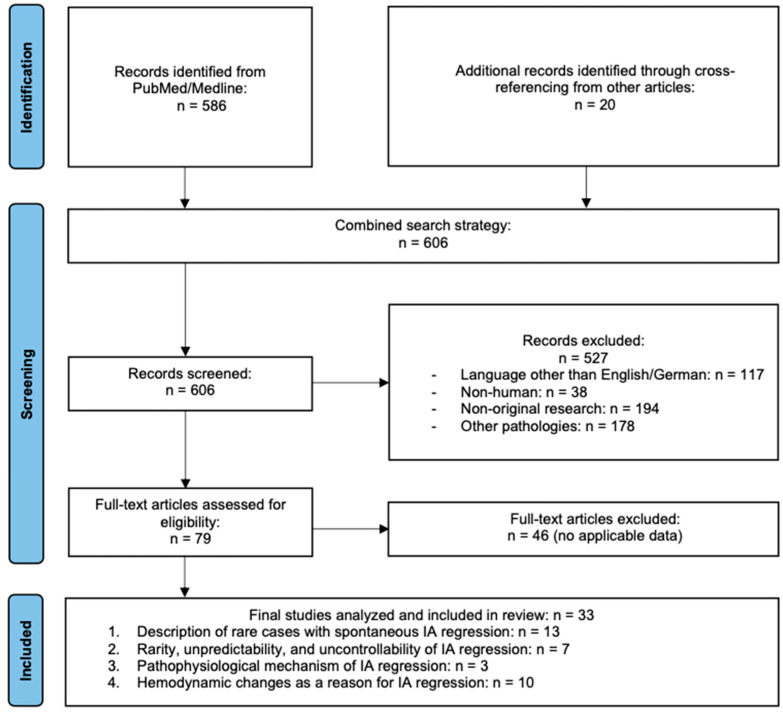
Prisma flowchart. Of n = 606 studies identified through the combined search strategy, finally, n = 33 papers were included in the literature review.

The systematic literature review was conducted in strict accordance with the PRISMA guidelines [14], focusing on the spontaneous thrombosis of IAs. A detailed search strategy was employed using the terms “intracranial aneurysm”, “spontaneous thrombosis”, “regression” and “occlusion”. The databases PubMed and Medline were searched for peer-reviewed articles published from 1951 to 2024. Articles that met the below listed inclusion criteria were included when they reported spontaneous IA thrombosis in humans, either following intervention (e.g., clipping) or without prior treatment in “native” IAs. All papers reporting the regression of a human IA with at least an abstract written in English or German language were included. Cross-referenced articles in the included papers were also included. Experimental studies on non-human IAs, papers on non-intracranial aneurysms, and non-original research such as conference papers, opinions, and letters were excluded. A list of keywords and synonyms for each concept was compiled and these keywords were combined using Boolean operators (AND, OR). The following search strategy was used on 18 January 2025: (“intracranial aneurysm”) AND (“thrombosis” OR “occlusion” OR “regression”) AND “spontaneous”. Additionally, filters for species (human), and publication date (from 1951 to 2024) were applied. Two authors independently reviewed the titles, abstracts, and full texts of 586 articles and 20 additional records identified through cross-referencing from other articles, with 33 meeting the inclusion criteria (Figure 1). Articles were categorized based on their focus, including spontaneous regression, unpredictability of IA regression, pathophysiological mechanisms, and hemodynamic changes responsible for spontaneous IA thrombosis (Table 1). Any discrepancy in study selection was discussed and decided upon consensus between the two authors (KC and BEG).

### 2.4. Case Report

#### Case Description

**Figure 2:**
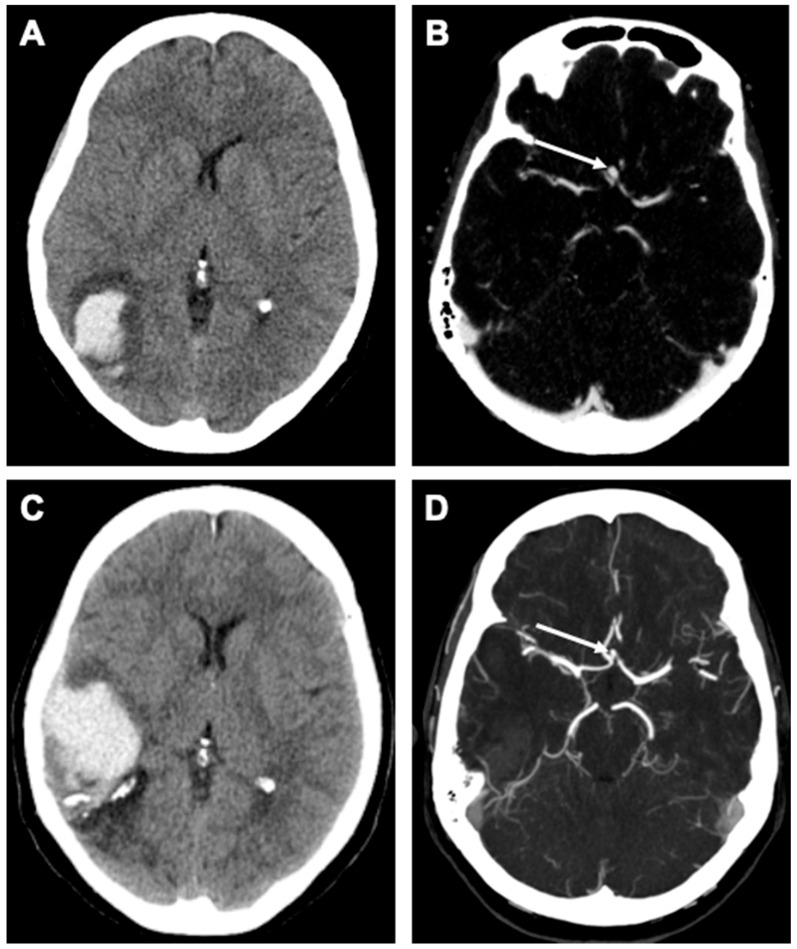
Illustrative panel showing intracranial hemorrhage unrelated to the IA. Initial CT scan (**A**) shows parieto-occipital hemorrhage on the right hemisphere, and the corresponding CT angiography (**B**) shows a saccular IA of the ACom artery (arrow). Nine years later, CT imaging reveals a recurrent hemorrhage in the right temporal region (**C**), while the previously detected IA has regressed in size (arrow in (**D**)).

**Figure 3:**
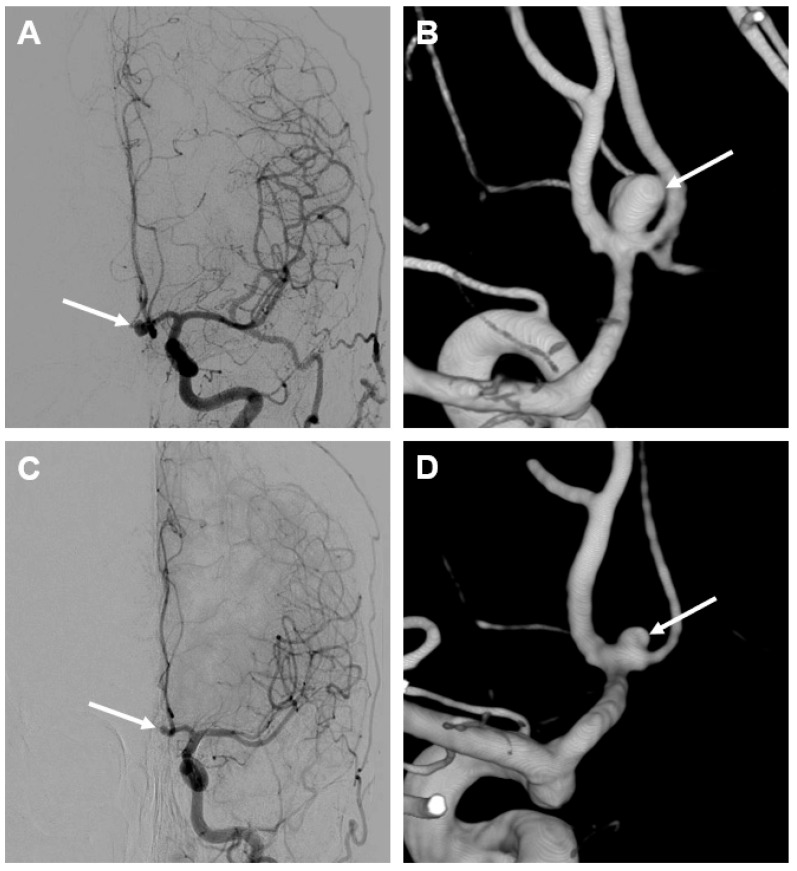
Illustrative example of spontaneous IA regression. Initial DSA (**A**) and the corresponding 3D reconstruction (**B**) show a saccular IA of the anterior communicating artery (arrow) with a size of 3 × 4 mm at the time of diagnosis. At the 9-year follow-up (**C**,**D**), the former IA visibly regressed (arrow) to a size of 2 × 2.5 mm without any interventions. Imaging did not reveal any signs of intra-aneurysmal thrombosis or wall calcification.

A 56-year-old female patient presented with an ischemic cerebrovascular event complicated by hemorrhagic transformation in the posterior MCA territory in the right hemisphere (Figure 2). During the diagnostic workup, an incidental anterior communicating artery (ACom) aneurysm was detected on a CT angiography (CTA). To complete the diagnostic evaluation, a digital subtraction angiography (DSA) was performed, which confirmed the presence of the ACom IA, measuring 3 × 4 mm (Figure 3). The IA was not related to the hemorrhage in any way. Given its asymptomatic nature and regular morphology, interdisciplinary discussion revealed a conservative management strategy, including regular FU (follow-up) examinations by means of non-invasive imaging.

At the time of the initial presentation, the patient had no known pre-existing conditions, no history of hypertension, and did not take any regular medication. Laboratory findings, including platelet count (245 G/L), hemoglobin (136 G/L), and hematocrit (0.385 L/L), were within normal ranges throughout the clinical course. Coagulation parameters were also normal (Quick 92%, INR 1). During the initial hospitalization, a patent foramen ovale (PFO) grade III was diagnosed. Given the ischemic lesions in the posterior circulation, a paradoxical embolism was suspected. The patient was started on antiplatelet therapy with aspirin and a statin. Additionally, she was prescribed levetiracetam after presenting with tingling paresthesias in her left arm.

A conservative management approach was proposed based on the patient’s preference and the presumed non-aneurysmal origin of the hemorrhage. Differential diagnoses included cerebral amyloid angiopathy (CAA) and her history of ischemic events. Follow-up imaging was recommended and the patient agreed with the proposed conservative strategy for the IA after detailed discussion. Unfortunately, she was lost to further FU.

Nine years after the initial ischemic event, the patient presented again with a sudden onset of severe headache, described as the worst headache of her life, accompanied by vomiting and fever. Upon arrival at the Emergency Department, the patient presented with a Glasgow Coma Scale (GCS) score of 14 (A3 V5 M6). Neurological examination revealed no focal deficits but a positive meningism. A CT scan revealed an atypical right temporal intracranial hemorrhage with ventricular extension and minimal subarachnoid blood. These features raised suspicion of cerebral amyloid angiopathy (CAA) or pial venous thrombosis. CTA ruled out sinus venous thrombosis, and the ACom aneurysm appeared unchanged in morphology, with no de novo IAs or additional vascular malformations detected. Subsequent imaging with MRA and DSA showed that the ACom aneurysm had regressed in size from the previous 3 × 4 mm to 2 × 2.5 mm (Figure 3) in the absence of an intra-aneurysmal thrombus. Importantly, the IA was again not identified as the source of the hemorrhage. The patient was admitted to the intensive care unit (ICU) for close neurological monitoring and, after 10 days, she was discharged to a rehabilitation facility. Interdisciplinary discussion concluded that the ACom aneurysm was unrelated to the intracranial hemorrhage, and the exact cause of the bleeding remained unclear, with a primary suspicion of CAA. Six months after the latest event, a follow-up DSA demonstrated a stable ACom aneurysm, unchanged in size and morphology (approximately 2 × 2.5 mm). One month later, a cranial MRA revealed the resorption of the subacute to chronic right temporal lobar hemorrhage, along with post-hemorrhagic encephalomalacia. Again, since no vascular abnormalities were detected, these findings supported the hypothesis of CAA as a possible underlying etiology for the hemorrhage.

### 2.5. Outcome and Follow-Up

The most recent follow-up, three years after the second bleeding event, with MRI/MRA confirmed the long-term stability of the ACom aneurysm, which remained unchanged in size and morphology, with no new IAs identified. Throughout this period, the patient remained neurologically stable, undergoing physiotherapy and occupational therapy to support recovery. She showed no focal deficits and required no surgical or endovascular intervention for the IA.

## 3. Results

### 3.1. Quantitative Summary of Included Cases

To improve pattern recognition across the literature, a summary of patient demographics, aneurysm characteristics, and regression intervals was generated (Table 2). Among the 33 included cases, patient age ranged from 13 to 75 years (mean of 47.2 ± 16.1), aneurysm sizes ranged from 2 mm to 25 mm (mean of 6.1 ± 4.9 mm), and follow-up durations ranged from 3 months to 13 years (median of 24 months). Most aneurysms were located in the anterior circulation, particularly at the anterior communicating (ACom) and middle cerebral arteries (MCAs). Regression mechanisms were inconsistently described and were variably attributed to spontaneous thrombosis, flow-related changes, or anatomical predisposition.

### 3.2. Literature Review

The literature overwhelmingly consists of retrospective studies and case reports, and none of the reviewed articles identified a predictive biomarker or specific early sign that could reliably indicate spontaneous IA regression. A systematic literature search was conducted according to PRISMA guidelines. A total of 606 articles were identified using database searches, and 20 additional records were included via cross-referencing. After screening and applying inclusion and exclusion criteria, 33 studies were included in the final review (see Figure 1 for PRISMA flowchart). To synthesize the data, studies were categorized into four primary themes, as also reflected in Table 1: (1) Description of rare cases with spontaneous IA regression—reports that document individual cases of IA thrombosis without intervention, including early historical descriptions. (2) Rarity, unpredictability, and uncontrollability of IA regression—literature emphasizing the unpredictability of this phenomenon and the necessity of careful follow-up. (3) Pathophysiological mechanisms of IA regression—studies proposing biological explanations, such as vasospasm or coagulation cascade alterations. (4) Hemodynamic changes as a reason for IA regression—articles linking regression to altered flow dynamics due to aging, vascular events, or surgical intervention. These categories helped to analyze the phenomenon from different perspectives, ranging from purely observational case reports to theoretical explorations of underlying mechanisms. Detailed characteristics of the included studies and their assignment to the respective categories are summarized in Table 1. Discussion and interpretation of the findings are presented in the subsequent section.

## 4. Discussion

This study reports an exceptional case of spontaneous regression of an ACom aneurysm over a nine-year period without surgical or endovascular intervention. Such regressions, though rare, underscore the dynamic and unpredictable behavior of IAs. Our systematic review identified 33 published cases (see Table 1), grouped into four categories: (1) rare spontaneous regressions, (2) unpredictability, (3) physiological mechanisms, and (4) hemodynamic factors.

The lack of prospective data significantly limits our ability to draw definitive conclusions or identify predictive markers for IA regression. Much of the early literature—dating from 1944 to the early 1980s—describes spontaneous thrombosis as a rare and largely unpredictable occurrence. Dandy [15] was among the first to report such cases, noting their exceptional nature and emphasizing the need for surgical treatment due to unpredictability. Epstein [16] and Lindgren [17] also reported MCA aneurysms that regressed without intervention, though only after extended follow-up. These and other early cases (e.g., Hemmer and Umbach [18], Spallone et al. [24]) laid the foundation for our understanding. More recent studies, such as those by Ueta [26] and Hassan [27], estimate that spontaneous IA thrombosis occurs in only 1–3% of cases.

### 4.1. Spontaneous Regression as a Rare and Unpredictable Phenomenon

Many reports have documented regression in small, narrow-necked IAs that may be predisposed to spontaneous thrombosis. Dandy [15] and Epstein [16] described some of the earliest known cases, while Lindgren [17] noted a spontaneously regressing MCA aneurysm over a period of three years. These reports are supported by Björkesten and Troupp [19], Schunk [20], and others who documented similar findings. Such regression often occurred without preceding intervention, though its underlying causes remained speculative. In our own clinical experience, only one comparable case was seen in over two decades. As summarized in Table 1 (category 1), these cases collectively affirm the rarity of regression and the absence of consistent predictors.

### 4.2. Rarity, Unpredictability, and Uncontrollability of IA Regression

Although rare, IA regression is not always permanent. Several authors describe cases where regression was followed by reappearance or recanalization. Scott and Ballantine [28] observed a giant MCA aneurysm that thrombosed and later reappeared on imaging. Spetzler et al. [8] also reported a case in which an IA vanished from angiograms, only to recur. Davila et al. [29] emphasized that favorable neurological status and imaging-confirmed regression were not sufficient for prediction. Tan [30] and Choi [31] each published cases of initially regressed aneurysms that later enlarged or reopened, highlighting the phenomenon’s uncontrollability. Kalin-Hajdu et al. [32] and Jun et al. [33] reinforced this uncertainty in both native and post-clipping aneurysms. Our patient’s ACom aneurysm regressed without evident hemodynamic or anatomical predispositions, further underscoring the phenomenon’s elusiveness (Table 1, category 2).

### 4.3. Pathophysiological Mechanisms Leading to IA Regression

Various mechanisms have been proposed to explain spontaneous IA thrombosis. Vasospasm and neck morphology are recurrent themes: Moritake et al. [35] linked vasospasm-induced flow restriction to thrombosis, particularly in narrow-necked aneurysms. Fodstad et al. [34] and Warschewske et al. [36] examined antifibrinolytic influences, including medication-induced changes in clotting dynamics. Our patient’s ACom aneurysm exhibited narrow-neck morphology and a history of hemorrhage, both of which could have promoted local clot formation. Ueta et al. [26] described thrombosis in a non-branching supraclinoid ICA aneurysm, and Hassan [27] reported a clipped ACA aneurysm remnant that spontaneously thrombosed. These studies, as well as Jun et al. [33], suggest that spontaneous thrombosis may also occur in surgically altered IAs. All examples point toward complex biological and structural factors contributing to regression (Table 1, category 3). While case reports provide foundational observations, an understanding of spontaneous regression requires the integration of basic science. Animal models, such as elastase-induced aneurysms in rabbits or rats, demonstrate that intra-aneurysmal thrombosis may be triggered by low wall shear stress (WSS), altered oscillatory shear index (OSI), and inflammatory cytokine activity [47,48]. These biomechanical environments promote endothelial dysfunction, neointimal thickening, and mural thrombosis. Computational fluid dynamics (CFD) studies further support that saccular aneurysms with narrow necks and flow stasis are prone to thrombogenesis. Future studies of regression phenomena should incorporate vessel wall imaging, CFD modeling, and histopathological correlation where possible.

### 4.4. Hemodynamic Changes as a Driver of Spontaneous Regression

Hemodynamic alterations are also implicated in spontaneous regression. Yeh et al. [39] and Cantore et al. [40] showed regression following EC-IC bypass or ICA occlusion, where flow redistribution likely reduced intra-aneurysmal perfusion. Tsimpas et al. [45] reported regression in a downstream MCA aneurysm after the treatment of a proximal IA with a flow diverter. These cases suggest that treating one aneurysm can impact others via hemodynamic shifts. Our patient underwent no vascular intervention, but gradual changes due to aging, vessel remodeling, or subtle shifts in cerebral perfusion may have played a similar role. Modern imaging techniques such as high-resolution MRI and DSA, as noted by Kim et al. [46] and others, enable a better assessment of such changes. The cases in Table 1, category 4, emphasize the need to consider systemic and flow-related factors when evaluating IA regression.

In summary, spontaneous IA regression remains a rare and unpredictable event. While individual cases may be associated with distinct anatomic, physiologic, or hemodynamic conditions, consistent predictive factors are lacking. Detailed documentation and long-term follow-up, supported by the literature as shown in Table 1, remain essential for understanding and managing this phenomenon.

## 5. Limitations

A limitation of this study is the reliance on retrospective data and case reports, which carry a risk of selection bias and inconsistent reporting standards. The relatively small number of cases reviewed (n = 33) represents the rarity of the condition but limits the generalizability of the findings. Additionally, the lack of long-term follow-up in many of the included studies makes it difficult to determine the durability and clinical significance of spontaneous regression. Future research addressing these gaps is essential for improving our understanding of this rare phenomenon. Prospective studies with standardized imaging protocols might help validate proposed mechanisms or identify predictive markers for IA regression.

## 6. Conclusions

The spontaneous regression of IAs, although rare and poorly understood, offers a unique insight into cerebrovascular dynamics and remodeling with significant implications for clinical management. This review proposes a standardized definition of regression and integrates both descriptive and mechanistic data. Hemodynamic factors, coagulation pathways, and vascular wall integrity appear central to the process, yet identifying high-risk patients remains challenging. Understanding these factors could improve diagnostic workflows, reduce unnecessary follow-ups, and lead to targeted therapeutic approaches, including non-invasive treatments. Ultimately, these insights may refine patient care, improve outcomes, and enhance decision-making for those at risk of aneurysmal subarachnoid hemorrhage. Advanced imaging technologies and ongoing research are essential to uncover the mechanisms behind regression and to develop effective monitoring and intervention strategies. Future research could employ longitudinal vessel wall MRI with black-blood sequences, CFD modeling to assess flow-related thrombotic risks, genetic profiling of extracellular matrix remodeling genes, and circulating biomarkers. Clinical monitoring protocols for regressed aneurysms should include MRA or CTA at 3 months post-regression and ongoing regular imaging thereafter.

## Figures and Tables

**Table 1:** Overview of studies reporting on IA regression. Studies can be summarized in four categories: First, those just reporting on IA regression as a rare phenomenon; second, those concluding that IA regression is rare, unpredictable, and uncontrollable; third, those suggesting a (patho-)physiological mechanism to explain the regression; and fourth, those suggesting hemodynamic changes to be the underlying reason for IA regression.

Year	Authors	Location and Number of IAs	Treatment	Peculiarity	Conclusion
(1) Description of rare cases with spontaneous IA regression					
1944	Dandy et al. [15]	Different IA locations	Clipping	Summary of different locations of IAs and their surgical accessibility.	Six surgical methods to possibly cure an IA, as spontaneous thrombosis occurs only rarely.
1953	Epstein et al. [16]	ACom, n = 1	None	Roentgenological demonstration of IA by pneumo-encephalogram.	Cerebral angiography as the most reliable method of diagnosis for the detection of IAs.
1958	Lindgren et al. [17]	MCA, n = 1	None	SAH patient.	Regression over three years.
1960	Hemmer and Umbach [18]	MCA/ posterior cerebral artery (PCA), n = 1	None	Visible IA two and nine weeks after hemorrhage. Not visible after six months.	Rarity of follow-ups in cases with arteriographically confirmed obliteration.
1962	Björkesten and Troupp [19]	Different IA locations, n = 25	Unsuccessful treatment by clipping	Two-year follow-up showed an occlusion of the IA.	Rare occurrence of spontaneous thrombosis of an IA after attempted clipping.
1964	Schunk et al. [20]	Literature review and case report, different IA locations	None	Contrast retention within IA in all three phases as a predisposing factor of thrombus formation.	Displacement of vessels adjacent to the partially thrombosed IA as an indicator of size and location of the thrombus.
1966	Lodin et al. [21]	PCom (posterior communicating artery), n = 1 Left superior cerebellar artery, n = 1	Clipping	Unsuccessful treatment by clipping.	Follow-up angiography in incidental and non-operated ruptured IA is justified, since they tend to increase in size.
1978	Edner et al. [22]	ACom, n = 1	None	Administration of dexa-methasone and EACA.	Impossibility of prediction of the spontaneous thrombosis of ruptured IA without intervention due to its rare occurrence.
1977	Scott and Garrido [23]	MCA, n = 1	Epsilon amino-caproic acid	Giant IA.	Spontaneous thrombosis of an IA is a rare event.
1981	Spallone et al. [24]	Supraclinoid ICA (internal carotid artery), n = 1	None	Spontaneous cure of an IA after recurrent SAH.	Rarity of spontaneous cure of IAs.
1983	Benedetti et al. [25]	ACom, n = 1	Clipping	Despite negative angiographic control, the patient was operated, and the aneurysmal sac was the parent.	An IA, revealed at operation, failed to opacify in the absence of vasospasm and thrombosis of parent vessels.
2004	Ueta et al. [26]	ICA, n = 1	None	First reported case of a ruptured IA at non-branching site of the supraclinoid ICA with complete spontaneous regression.	Spontaneous regression of IA can occur even at non-branching sites without intervention or treatment.
2009	Hassan et al. [27]	Anterior cerebral artery (ACA), n = 1 SAH	Clipping	Spontaneous thrombosis of a recurrent clipped IA.	Spontaneous thrombosis of an IA remnant may occur even without further treatment.
**(2)** **Rarity, unpredictability, and uncontrollability of IA regression**					
1972	Scott and Ballantine [28]	MCA, n = 1	None	Progressive thrombosis of a giant IA and the underlying MCA branch.	Probably non-operative treatment should be preferred.
1974	Spetzler et al. [8]	Left frontopolar artery, n = 1	Clipping	Disappearance and reappearance of an IA.	Terms such as “complete” and “spontaneous” cure must be used with caution, since the natural history of IA is not understood yet.
1984	Davila et al. [29]	ACom, n = 1	None	Spontaneous thrombosis of an IA.	Three conditions necessary for spontaneous cure: (1) favorable neurological status, (2) complete disappearance of the IA on imaging, and (3) confirmation of its regression without intervention.
2001	Tan et al. [30]	MCA, n = 1	Clipping	Re-clipping of a remnant five months post initial operation. Further regrowth due to an unsuccessful bypass.	Unpredictability of growth and regression of IAs.
2010	Choi et al. [31]	MCA, n = 1	Wrapping using pericranium and surgical glue	Stability for one year, regression after three years.	Recanalization may occur also in completely thrombosed IA, with possibility of growth or rupture.
2011	Kalin-Hajdu et al. [32]	MCA, n = 1	None	Angiography on scheduled embolization revealed that the IA had already undergone complete thrombosis.	Spontaneous occlusion and regression of a IA is exceptional and unpredictable.
2016	Jun et al. [33]	ACom, n = 1	Clipping	Reluctance from the patient regarding reoperation.	Absence of a reliable methods to predict whether an IA remnant will spontaneously regress or grow.
**(3)** **Pathophysiological mechanism of IA regression**					
1979	Fodstad et al. [34]	MCA, n = 1	AMCA (tranexamic acid)	Spontaneous thrombosis might occur more often when anti-fibrinolytic drugs are administered.	Possible local inhibition of plasminogen activators in and around the IA wall when using AMCA.
1981	Moritake et al. [35]	PCom, n = 1	None	Ruptured IA.	Crucial roles of vasospasms and relative narrowness of the aneurysmal neck in cases of spontaneous IA thrombosis.
1999	Warschewske et al. [36]	Anterior cerebral artery (ACA), n = 1	None	Spontaneous thrombosis of a giant IA.	Spontaneous thrombosis may occur under certain anatomic and hemodynamic conditions. Conservative management and follow-up examinations in asymptomatic patients is suggested.
**(4)** **Hemodynamic changes as a reason for IA regression**					
1957	Marguth et al. [37]	ICA, n = 1	None	Spontaneous SAH at age thirteen yeats, regression on control thirteen years later.	Fully visible IA sac in the arterial phase, only contrast residuals visible in the early and late venous phases.
1964	Höök and Norlen [38]	MCA, n = 1	Ligation of the aneurysmal neck	Impossibility of contrast filling of the carotid artery through an acute cerebral edema.	Intracranial treatment methods for IA are more effective and should be preferred.
1997	Yeh et al. [39]	Terminal ICA, n = 1	EC–IC bypass	Spontaneous thrombosis of the IA at two-year follow-up.	Hemodynamic changes in the blood flow of the parent artery after EC–IC bypass caused obliteration.
1999	Cantore et al. [40]	Supraclinoid ICA, n = 2	Clipping/ Bypass	Postoperative angiography demonstrated spontaneous occlusion of the IAs.	Spontaneous occlusion of an IA may be induced or favoured by hemodynamic vascular alterations that take place inside the IA after a high-flow EC-IC bypass has been created.
2000	Senn et al. [41]	PCom, n = 1	Carotid end-arterectomy (CEA)	Regression after ipsilateral carotid endarterectomy.	Formation and regression of the ‘flow-related’ IA was associated with hemodynamic changes in blood flow of the right posterior communicating artery (PCoA) and the right ICA.
2004	Hans et al. [42]	ICA, n = 3 (1 giant cavernous ICA aneurysm, 2 supra-ophthalmic IAs)	Endo-vascular closure	Spontaneous regression of two supraophthalmic IAs following flow pattern alteration by proximal ICA IA treatment.	Changes in cerebral hemodynamics might potentially lead to plastic changes in the vessel architecture, therefore IAs can be flow related.
2005	Chow et al. [43]	Paraclinoidal ICA, n = 2	Endovascular treatment	Disappearance of an ICA aneurysm after endovascular treatment of a concurrent ipsilateral ICA aneurysm.	Hemodynamic alterations may contribute to regression.
2012	Li et al. [44]	ICA, n = 1	Carotid end-arterectomy (CEA)	Spontaneous regression of an intracranial IA after carotid endarterectomy.	Regression and potential formation of IAs may correlate with hemodynamic factors associated with stenosis of the contralateral ICA.
2015	Tsimpas et al. [45]	ICA, n = 3 right MCA, n = 1 left MCA, n = 1	Coiling/ Flow Diverter	Regression of a downstream M2-aneurysm after Flow Diverter treatment of ICA aneurysms.	Changing flow dynamics of a proximal vessel may eventually lead to spontaneous regression of a downstream IA.
2018	Kim et al. [46]	Right anterior temporal artery, n = 1	Clipping	Regression after contralateral MCA clipping.	Spontaneous occlusion may occur even without direct treatment. High-resolution MRI may provide insights into morphology and IA changes over time.

**Table 2:** Quantitative summary of key metrics.

Patient Age	Aneurysm Location	Aneurysm Size	Follow-Up Duration
56 y/o	ACom	3 × 4 mm (diagnosis) 2 × 2.5 mm (FU)	9 years

## Data Availability

Data supporting the findings of this study are available upon reasonable request from the corresponding author. The data are not publicly available due to privacy restrictions.

## Abbreviations

The following abbreviations are used in this manuscript:

ACom	anterior communicating artery
CT/A	computed tomography angiography
CFD	computational fluid dynamics
DSA	digital subtraction angiography
EC-IC	extracranial–intracranial
FU	follow-up
IA	intracranial aneurysm
ICA	internal carotid artery
iDSA	intraoperative digital subtraction angiography
MCA	middle cerebral artery
MRI/MRA	magnetic resonance imaging / angiography
PCom	posterior communicating artery
SAH	subarachnoid hemorrhage
